# A Unique Case of Appendiceal Intussusception (Inversion): A Case in Bloom

**DOI:** 10.3390/diagnostics14050555

**Published:** 2024-03-06

**Authors:** Stylianos Mantalovas, Eleni Paschou, Ismini Kountouri, Christina Sevva, Konstantinos Papadopoulos, Panagiota Roulia, Marios Dagher, Styliani Laskou, Vasileios Lagopoulos, Charilaos Koulouris, Fedra Louloudopoulou, Periklis Kopsidas, Konstantinos Sapalidis, Isaak Kesisoglou, Christoforos Kosmidis

**Affiliations:** Third Surgical Department, “AHEPA” University Hospital, Medical Faculty, Aristotle University of Thessaloniki, 1 Kiriakidi Street, 54636 Thessaloniki, Greece; steliosmantalobas@yahoo.gr (S.M.); i.kountouri531@gmail.com (I.K.); christina.sevva@gmail.com (C.S.); kostaspap1995@hotmail.com (K.P.); panagiotar96@gmail.com (P.R.); mariosdag@gmail.com (M.D.); stelaskou@gmail.com (S.L.); vaslag@gmail.com (V.L.); charilaoskoulouris@gmail.com (C.K.); faidraaloulou@gmail.com (F.L.); periklis.kop@gmail.com (P.K.); sapalidiskonstantinos@gmail.com (K.S.); ikesis@hotmail.com (I.K.); dr.ckosmidis@gmail.com (C.K.)

**Keywords:** acute, appendicitis, appendiceal, intussusception, inversion, abdominal, pain, case report

## Abstract

A 40-year-old female patient presented to a secondary facility with dull lower abdominal pain and a persistent low-grade fever. Her laboratory results showed elevated inflammation markers. A CT scan revealed two abscesses in the lesser pelvic region in direct contact with the apex of the appendix, the posterior wall of the uterus, and the right-side appendages. The patient responded well to intravenous antibiotics, and an MRI scan revealed the cause to be an appendiceal rupture. The patient was scheduled for an appendectomy. The procedure started laparoscopically but had to be converted to an open one with a midline infra-umbilical incision in order to protect the right appendages. A standard appendectomy was conducted, and the histology report revealed rupture of the appendix with concomitant wall inversion in the context of fibrous adhesions as well as obstruction due to a fecalith. Patient recovery and follow-up were excellent. Acute appendicitis, while frequently encountered in surgical practice, can present a diagnostic conundrum when it manifests in an atypical manner. This unique form of inversion appeared to confer a protective role against peritonitis, primarily through the mechanism of obstruction occurring centrally to the rupture. We suggest that this case should be included in current classifications as a partial inversion of the appendix after rupture and inflammation.

While appendicitis typically presents with right iliac fossa pain, conditions such as retrocecal appendicitis, plastron appendicitis, Crohn’s disease, mucocele of the appendix, adenocarcinoma, carcinoid tumor of the appendix, endometriosis, and finally the very rare case of intussusception or inversion of the appendix complicate both the differential diagnosis and management of the patient [1,2]. Appendiceal inversion happens when part of the appendix invaginates into the adjoining intestinal lumen, causing obstruction, and it ranges from complete to partial inversion, with the latter usually being an incidental finding in the context of colonoscopy [2]. In most cases, however, appendiceal intussusception manifests as acute appendicitis (78%), bowel obstruction (26%), and finally hematochezia (23%). Several classifications have been proposed to enhance understanding of these conditions, which is crucial for evidence-based management of similar cases [3]. The classification by Forsall et al. includes almost all possible categories and has undergone contemporary modifications, such as those of Park et al. [3,4,5]. The fundamental idea underlying the classification system involves the correlation between inflammation and intussusception occurring at the base of the cecum.

A 40-year-old female patient with a history of hysteroscopic myomectomy was transferred to a tertiary facility from a secondary hospital due to diagnostic challenges involving suspected plastron appendicitis and pelvic inflammation. The patient presented with a persistent low-grade fever and localized abdominal pain, predominantly at Lanz’s point rather than McBurney’s point, without symptoms of anorexia, vomiting, or peritonitis. Laboratory investigations revealed a white blood cell count (WBC) of 12.02 × 109 cells/L with 80.1% neutrophils (Neut) and a C-reactive protein level of 25.2 mg/L. A computed tomography (CT) scan revealed two abscesses in the lesser pelvic region, with the larger one measuring 5 × 10^−2^ m, as shown in Figure 1. These abscesses were adjacent to the appendix apex, posterior uterine wall, and right-side appendages.

Interventional radiologists were unable to drain the abscess cavity due to its small size and interference from adjacent bowel loops. Conservative treatment with intravenous antibiotics and fluids led to a complete resolution of fever and significant improvement in inflammatory markers within 48 h. The patient was discharged, and a pelvic MRI conducted one month later confirmed appendiceal rupture with abscess formation.

The decision was made to perform an elective laparoscopic appendectomy. However, due to the appendix’s proximity to the ovary and posterior uterine wall, the procedure had to be converted to an open one with a midline infra-umbilical incision. A sharp dissection of the appendix was conducted while preserving the integrity of the right fallopian tube and ovary. Careful, thorough cleaning of the area was achieved with compartmentalization of the abscess cavity by soaked surgical gauges. Within the abscess, apart from a small amount of pus, its content was mainly mucus, so the suspicion of mucocele was also raised.

Gross findings are shown in Figure 2. Our case is closer to type 1C, according to Park’s classification. The histological examination revealed a wall inversion in the context of fibrous adhesions. This condition may have resulted from a potential wall rupture caused by fecalith obstruction as shown in Figure 3. The patient was discharged 48 h after surgery, and her clinical progression during the postoperative follow-up was deemed excellent.

In addition to the usual types of appendiceal inversion, there is also the case of partial inversion in the context of rupture and inflammation. Inflammation is not the most common cause of appendiceal inversion in adults [4]. Inversion due to inflammation and rupture may act protectively due to obstruction centrally to the rupture. Yet when the inversion approaches the base of the cecum, the situation becomes more complex. It is imperative for the surgeon to possess comprehensive knowledge regarding all potential categories of appendiceal intussusception, so that even intra-operatively, one can make the right decision and perform a more extensive surgical intervention, such as when there is a precarious residual stump or when local conditions arise where the intussusception is close to the base, in order to avoid intussusception recurrence [6,7]. In order to prevent continuous intussusception following appendectomy, partial cecectomy is recommended for Types 1B, 1E, and 2 of the Park classification, while for Type 3, removal of the trigger point is advised. Hence, it is widely recommended to perform a wider resection from the outset, including the cecum in these cases [3,8].

In the present case, epithelial lining was identified on the external surface of the incisions without a clear margin of transition, which, on the basis of both microscopic and macroscopic images, was attributed to eversion of the appendix due to adhesions. This epithelial tissue was not endometrial, and intracellular mucus was not identified. Moreover, there were no dysplastic lesions. On the contrary, an obstruction of the appendiceal lumen was revealed centrally: small fecaliths. It should be emphasized that the base of the appendix and cecum were healthy, and the apex showed a large flexure. On the basis of the macroscopic and especially the microscopic information, this was clearly a common case of appendiceal inflammation that progressed to rupture due to obstruction. The rupture resulted in a plastron involving the corresponding internal genitalia. Due to the chronic nature of the case, the epithelial mucosa also occupied the outer layers of the appendix, giving this case of intussusception an almost “flower-like” appearance. Thus, this intussusception of the mucosa as well as the central obstruction retained and prevented the excretion of fecal contents into the free peritoneal cavity. Consequently, the contents of the abscess primarily consisted of pus and a small quantity of mucus, which originated from the inverted mucosal epithelium of the appendix. The presence of the inverted mucosa played a partially protective role, given the fact that it was far from the base of the cecum. Thus, this is a case of a partial inversion of the appendix after obstruction and rupture, with concurrent plastron creation. It is also considered rare that the inversion was a product of inflammation alone, since the most common cause of appendiceal inversion in adults is endometriosis (23%), followed by mucocele (19%) and inflammation (19%) [4]. Malignant lesions associated with appendiceal inversion are also seen in many cases [4]. In this particular case, appendectomy is the appropriate procedure, and it is recommended to be performed laparoscopically, provided there are no contraindications [3,9]. We therefore suggest that this case should also be reported in current classifications as a partial inversion of the appendix after rupture and inflammation.

## Figures and Tables

**Figure 1:**
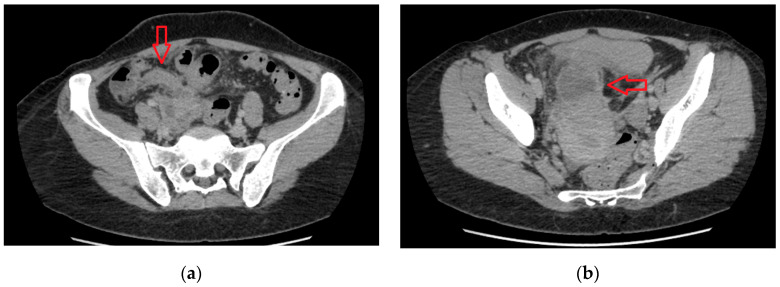
(**a**) The appendix is shown with the arrow. Following its course from the base at the cecum, a possible rapture point can be seen at the apex, while (**b**) shows a 5 × 4.2 × 10^−2^ m pelvic abscess [arrow] that displaces the bladder anteriorly and the uterus.

**Figure 2:**
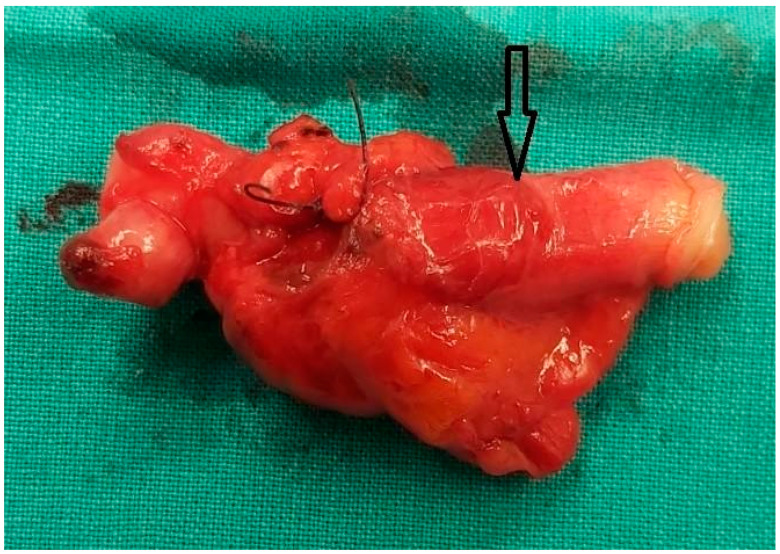
Gross findings show adhesions close to the apex of the appendix, rupture of its wall centrally, and an appendiceal intussusception. The external location of the epithelium is indicated by the arrow.

**Figure 3:**
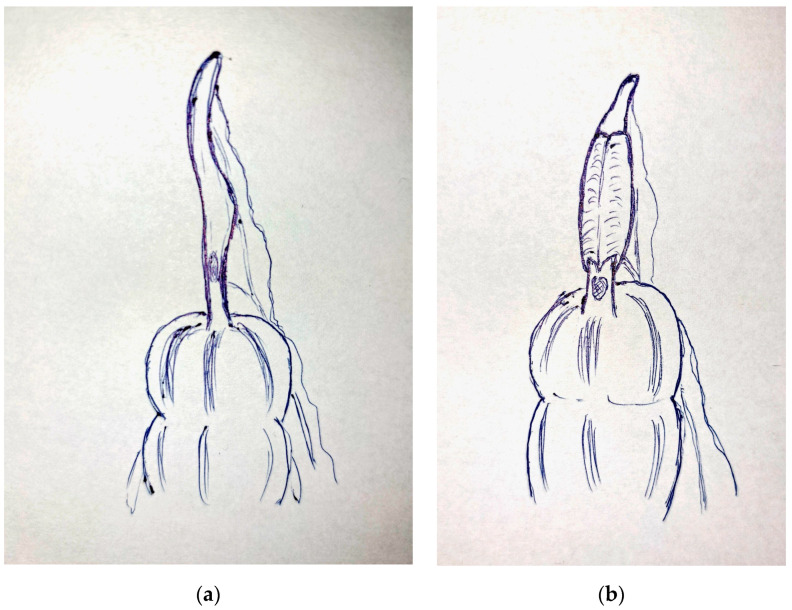
Our case: (**a**) The appendix with feacalith obstruction before rupture. (**b**) After inversion of the mucosa (intussusception) and rupture distally to the intussusception, giving it a unique “flower-like” appearance.

## Data Availability

All data used in this case report is available from the corresponding author and can be provided upon reasonable request.
